# Base Excision DNA Repair in Plants: *Arabidopsis* and Beyond

**DOI:** 10.3390/ijms241914746

**Published:** 2023-09-29

**Authors:** Inga R. Grin, Daria V. Petrova, Anton V. Endutkin, Chunquan Ma, Bing Yu, Haiying Li, Dmitry O. Zharkov

**Affiliations:** 1 Siberian Branch of the Russian Academy of Sciences Institute of Chemical Biology and Fundamental Medicine, 8 Lavrentieva Ave., Novosibirsk 630090, Russia; dpetrova@niboch.nsc.ru (D.V.P.); aend@niboch.nsc.ru (A.V.E.); 2 Department of Natural Sciences, Novosibirsk State University, 2 Pirogova St., Novosibirsk 630090, Russia; 3 Engineering Research Center of Agricultural Microbiology Technology, Ministry of Education, Harbin 150080, China; chqm0913@163.com (C.M.); ybgirl1234@sina.com (B.Y.); lvzh3000@sina.com (H.L.); 4 Heilongjiang Provincial Key Laboratory of Plant Genetic Engineering and Biological Fermentation Engineering for Cold Region, Harbin 150080, China; 5 School of Life Sciences, Heilongjiang University, Harbin 150080, China

**Keywords:** DNA damage, DNA repair, base excision repair, plants

## Abstract

Base excision DNA repair (BER) is a key pathway safeguarding the genome of all living organisms from damage caused by both intrinsic and environmental factors. Most present knowledge about BER comes from studies of human cells, *E. coli*, and yeast. Plants may be under an even heavier DNA damage threat from abiotic stress, reactive oxygen species leaking from the photosynthetic system, and reactive secondary metabolites. In general, BER in plant species is similar to that in humans and model organisms, but several important details are specific to plants. Here, we review the current state of knowledge about BER in plants, with special attention paid to its unique features, such as the existence of active epigenetic demethylation based on the BER machinery, the unexplained diversity of alkylation damage repair enzymes, and the differences in the processing of abasic sites that appear either spontaneously or are generated as BER intermediates. Understanding the biochemistry of plant DNA repair, especially in species other than the *Arabidopsis* model, is important for future efforts to develop new crop varieties.

## 1. Introduction: BER Essentials

DNA repair is a vital process in all living organisms [1]. Repair is required to restore the integrity of DNA after damage that, in human cells, occurs at the level of tens to hundreds of thousands of events per cell, per day [2,3]. The most important sources of DNA damage are endogenous, including oxidative metabolism, spontaneous hydrolytic reactions, and replication machinery errors, whereas environmental factors, such as ionizing and UV radiation, and genotoxic biogenic or industrial chemicals, also contribute significantly [1]. In humans and higher animals, inactivation of repair systems is lethal or causes severe morbidity [1]. The accumulation of damage not eliminated by DNA repair, in many cases, leads to mutations and is among the primary causes of aging, carcinogenesis, and some neurodegenerative and autoimmune diseases [4,5].

**Figure 1:**
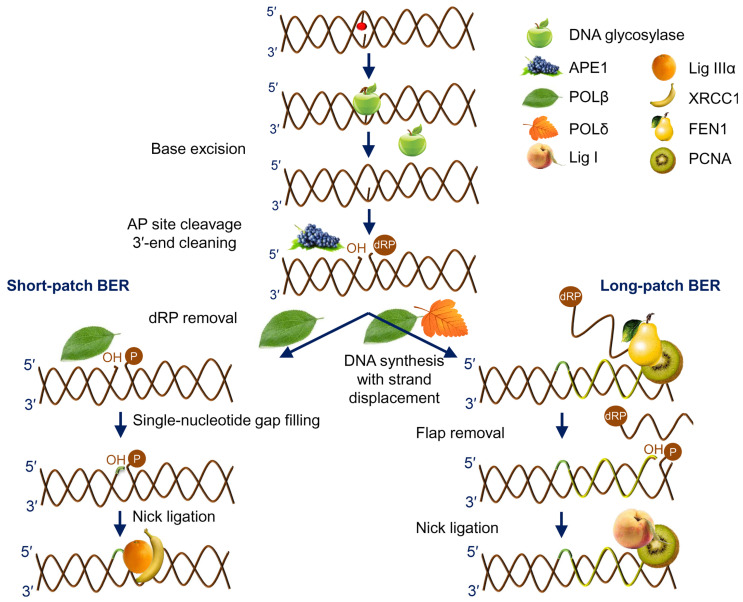
General scheme of short- and long-patch BER.

**Figure 2:**
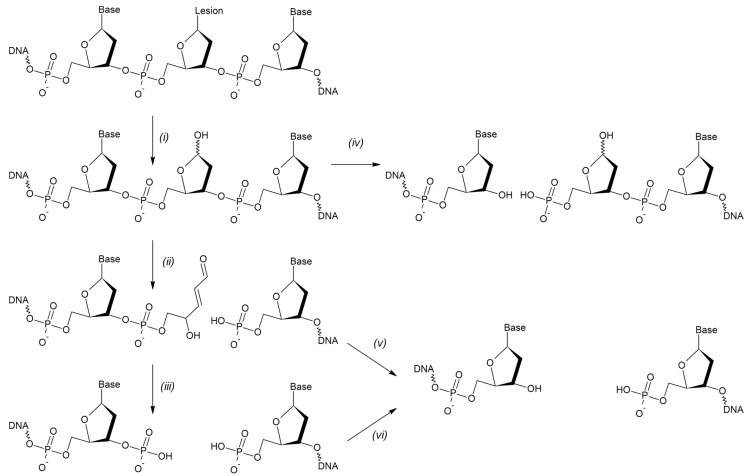
Chemistry of the initial BER steps. Only one DNA strand is shown for clarity. Monofunctional DNA glycosylases only hydrolyze the *N*-glycosidic bond (**i**), whereas bifunctional DNA glycosylases possess the AP lyase activity that catalyzes β-elimination (**ii**) or β,δ-elimination (**iii**). After the action of monofunctional glycosylases, AP endonucleases cleave the phosphodiester bond 5′ to the AP site (**iv**). After β-elimination, the 3′-phosphodiesterase activity of AP endonucleases removes the 3′-terminal α,β-unsaturated aldehyde (**v**). After β,δ-elimination, the 3′-phosphatase activity, either standalone or residing in AP endonucleases, removes the 3′-phosphate (**vi**).

The vast majority of intrinsic DNA lesions (products of spontaneous deamination, hydrolysis, oxidation, and metabolic-related alkylation) are dealt with by a system known as base excision repair (BER) [6,7,8]. The current model of BER (Figure 1) is mostly derived from studies in mammalian cells, with significant input from *E. coli* and yeast systems. If base damage is present in the genome, it is recognized and excised by DNA glycosylases, lesion-specific enzymes that hydrolyze the *N*-glycosidic bond in nucleotides. Eleven DNA glycosylases with different substrate specificities are presently known in humans, and eight in *E. coli* (Table 1), while some bacteria and viruses possess a few additional DNA glycosylases absent from these two species. Some DNA glycosylases only cleave the *N*-glycosidic bond, leaving a baseless 2′-deoxyribose (apurinic/apyrimidinic site, or AP site) in DNA (Figure 2). Others, termed DNA glycosylases/AP lyases, can nick DNA 3′ to the nascent AP site by β-elimination, leaving an α,β-unsaturated aldehyde at the 3′-end of DNA at the break. A subset of those (β,δ-lyases) proceeds even further, catalyzing the removal of the whole sugar fragment and leaving a single-nucleotide gap flanked by two phosphate groups (Figure 2). All DNA glycosylases/AP lyases share a common reaction mechanism and form a covalent Schiff base intermediate with C1′ of the damaged nucleotide in the reaction. Both β- and β,δ-elimination products at the 3′-end cannot be extended by DNA polymerases and need additional processing to expose a 3′-OH group. Thus, a second BER step involves hydrolytic enzymes that nick DNA at a phosphate 5′ to an AP site, or an unsaturated aldehyde, or a 3′-phosphate (Figure 2). The respective activities are referred to as AP endonuclease, 3′-phosphodiesterase, or 3′-phosphatase, although they usually reside within the same protein, which therefore can continue the process after any DNA glycosylase (Table 1). A notable exception is human APE1, which lacks a 3′-phosphatase activity and, thus, polynucleotide kinase/3′-phosphatase (PNKP) substitutes for APE1 after the action of human β,δ-lyases (NEIL1, NEIL2, and NEIL3).

After a free 3′-OH end is formed, a DNA polymerase can incorporate an undamaged dNTP complementary to the base in the template (Figure 1). In human cells, DNA polymerase β (POLβ) is the principal BER polymerase, although DNA polymerase λ (POLλ), the main non-homologous end joining polymerase, can be involved in specific cases, and DNA polymerase γ (POLγ) does the job in mitochondria. If the repair started with a monofunctional DNA glycosylase followed by an AP endonuclease, a 2′-deoxyribo-5′-phosphate (dRP) remnant lingers at this point at the 5′-end of DNA downstream of the nick, and POLβ polymerase activity is coupled with a dRP lyase activity that removes this moiety. One or several nucleotides can be incorporated (short-patch or long-patch BER, respectively; Figure 1), with a possible switch from POLβ to DNA polymerases δ or ε assisted by the PCNA processivity factor. If several nucleotides are replaced, the hanging flap is clipped by flap endonuclease, FEN1. In *E. coli*, DNA polymerase I is the major BER polymerase, and its 5′→3′ exonuclease activity degrades the downstream fragment. Finally, DNA ligase (LigI or LigIIIα in human cells, LigA in *E. coli*) seals the last nick restoring the integrity of DNA (Figure 1).

## 2. DNA Damage Specifics in Plants

Compared with animals, and probably more in common with bacteria, plants are exposed to an even wider variety of genotoxic stresses. Abiotic factors, such as light regime, temperature, and water availability, have a stronger impact on the biochemistry of plant cells compared with multicellular animals. While mitochondria are the sole major source of reactive oxygen species (ROS) in animal cells, chloroplasts generate an abundant additional ROS flow in plants [9,10]. Last, but not least, plants produce a wide variety of genotoxic secondary metabolites, of which psoralens, topoisomerase inhibitors (camptothecins, podophyllotoxins), and aristolochic acid are probably the best-studied examples [11,12,13,14,15]. Moreover, some widespread plant protein toxins, such as ribosome-inactivating proteins, also damage DNA on top of their primary mode of action [16]. To protect against abiotic stress associated with environmental factors, different repair systems operate in different plant organs, and the consequences caused by their deficiency can vary from a decrease in the stability of the seed genome to a decrease in the yield of adult plants [17,18].

Abiotic stress plays a critical role in plant life and is one of the main factors limiting crop productivity. Different types of abiotic stress can have a damaging effect on the genome of plant cells, both directly and through the production of reactive secondary metabolites and signaling molecules [19]. BER activation, double-strand break response, and nucleotide pool sanitization are essential elements of the abiotic stress response in plants [20,21,22]. Information on the role of plant BER in response to abiotic stresses other than known DNA damaging factors (such as ionizing radiation, UV radiation, or exposure to genotoxic compounds) is extremely limited, with the exception of BER-dependent epigenetic regulation [23,24]. However, some observations indicate that, even apart from the epigenetic mechanism, BER is indeed involved in protecting plants from thermal and osmotic stress [25,26,27,28]. This may be due, in part, to the fact that many types of abiotic stress lead to increased leakage of DNA-damaging reactive oxygen species from chloroplasts, and DNA repair may be needed to protect cells from this damage [29,30]. Characterization of the role of BER and other types of DNA repair in plant responses to abiotic stress is important for understanding the mechanisms of plant adaptation to adverse environmental conditions.

## 3. Plant DNA Glycosylases: The Universal Themes

### 3.1. Uracil–DNA Glycosylases

Uracil–DNA glycosylase (Ung in bacteria, UNG in humans) was the first DNA glycosylase discovered and studied with respect to its mechanism [31,32]. Several other DNA glycosylases have U as their target in DNA, namely mismatched uracil–DNA glycosylase (Mug) in *E. coli*, G/T mismatch-specific thymine DNA glycosylase (TDG), single-strand-selective monofunctional uracil-DNA glycosylase 1 (SMUG1), and methyl-CpG binding domain 4-containing DNA glycosylase (MBD4) in humans. Of these, Ung/UNG and SMUG1 are general-purpose repair glycosylases removing U from any DNA context, MBD4 is specialized for U repair in damaged CpG dinucleotides, and TDG mainly participates in active demethylation rather than in DNA repair [33,34]. With the exception of MBD4, all uracil–DNA glycosylases belong to the same structural superfamily, sharing the α/β-fold and a common evolutionary origin [35].

The first plant protein with the uracil–DNA glycosylase (or, indeed, any DNA glycosylase) activity was purified in 1983 from wheat germ and showed properties fairly typical for *E. coli* and human UNG [36]. Enzymatic activity removing U from DNA was detected in living cells or cell extracts from a number of phylogenetically diverged plant species [37,38,39,40]. *A. thaliana* cell extracts support the full BER cycle for U with base excision, AP site processing, dNMP incorporation, and nick ligation [41,42]. The same capability was demonstrated for a single-cell green alga, *Chlamydomonas reinhardtii* [43].

Cloned recombinant AtUNG removes U from both single- and double-stranded DNA, with a moderate preference for U:G pairs [42]. AtUNG seems to be the only enzyme capable of U excision in *Arabidopsis* since the *UNG* gene knockout totally eliminates the activity in cell extracts and enhances resistance to 5-fluorouracil, a chemical that promotes DNA instability via excessive U accumulation and excision [42]. Paradoxically, however, *ung* plants do not accumulate the expected C→T mutations, suggesting the presence of alternative repair mechanisms [44].

Genome sequencing reveals no orthologs of human TDG and SMUG1 in flowering plants, although a TDG homolog is found in green algae and a SMUG1 homolog is present in mosses, ferns, horsetails, and gymnosperms. At the sequence level, they are more closely related to bacterial rather than to metazoan homologs, indicating possible horizontal transfer events. None of these homologs are functionally characterized.

MBD4 in vertebrates, first discovered as a protein containing an MeCP2-like mC-binding domain, is thought to counteract deamination specifically in CpG dinucleotides [45,46,47]. The name of the plant homolog is somewhat of a misnomer, since plant MBD4 contains no methyl-binding domain [48,49]. However, the overall organization of AtMBD4 and hMBD4 is similar, with the C-terminal catalytic domain and an extended N-terminal part, with the catalytic residues fully conserved [48,49]. AtMBD4 excises U and, less efficiently, T mispaired with G, and also processes 5-BrU, 5-FU, 5-hydroxymethyluracil, and 5-hydroxyuracil, with a modest twofold preference for a 5′-XG-3′ context and no methylation status preference [48]. Since CG, CHG, and CHH sites are all widely used for methylation in plants, these properties are consistent with a view of AtMBD4 as a guardian of methylation sites against C deamination. Interestingly, truncation of the N-terminal part, leaving only the catalytic domain, improves the kinetics of AtMBD4 [48]. This finding may be of biological relevance, since of several functional AtMBD4 isoforms detected in vivo at the mRNA level, one splices out a cryptic intron and lacks a 116 amino acid fragment from the N-terminal part [49,50]. This protein isoform is directed to the nucleolus, whereas the longer one is evenly distributed in the nucleus, and the alternative splicing and relocalization is heat induced, underscoring the role of plant MBD4 in the abiotic stress response [50].

### 3.2. Oxidative Damage Repair

Oxidative DNA base damage presents the largest group of chemically diverse lesions, unavoidable in living organisms with an oxygen-based metabolism [51,52]. Most of them are cytotoxic, but some are miscoding; a textbook example of the latter is 8-oxoguanine (8-oxoG), which can pair with either C or A during replication and cause G→T transversions in the latter case [53]. High frequency and mutagenicity 8-oxoG necessitate a dedicated system for its repair, the so-called GO system, which consists of three enzymes, namely 8-oxodGTPase MutT/MTH1 (in *E. coli* and humans, respectively) that sanitizes the dNTP pool and prevent 8-oxoG incorporation by DNA polymerases, and two DNA glycosylases, Fpg/OGG1 and MutY/MUTYH (Table 1). Fpg and OGG1 are entirely different with respect to their sequence and structure, yet their substrate specificities are almost identical: they remove 8-oxoG from pairs with C and strongly discriminate against 8-oxoG:A substrates. As such, mispairs are most often formed when replicative DNA polymerases incorporate A opposite to 8-oxoG, an excision of 8-oxoG would replace it with T and finalize the mutation. So instead of Fpg/OGG1, 8-oxoG:A is recognized by MutY/MUTYH, which remove A and give repair polymerases (PolI in *E. coli*, POLλ in humans) a chance to incorporate C opposite to 8-oxoG and hand the baton to Fpg/OGG1 for the second round of repair (reviewed in [53,54]). In addition to 8-oxoG, Fpg and OGG1 excise formamidopyrimidines (Fapy), the products of hydroxyl radical attack at A or G, followed by reduction. The repair of oxidatively damaged pyrimidines is less complicated and proceeds essentially according to the general BER scheme; the involved enzymes are Nth and Nei in *E. coli* and NTHL1, NEIL1, NEIL2, and NEIL3 in human cells. One important distinction regarding the repair mechanism is that MutY/MUTYH, OGG1, and Nth/NTHL1 belong to the helix–hairpin–helix (HhH) structural superfamily and are either monofunctional (MutY/MUTYH) or catalyze β-elimination (OGG1 and Nth/NTHL1), whereas Fpg, Nei, and NEIL1–NEIL3 come from the helix–two-turn–helix (H2TH) structural superfamily and catalyze β,δ-elimination [55,56].

Plants possess a collection of all glycosylases for oxidative damage repair. A homolog of bacterial Fpg was cloned from an *A. thaliana* cDNA library based on sequence homology and termed MMH-1 (MutM Homolog 1; historically, MutM is an alternative name for Fpg) [57]. The primary transcript is alternatively spliced producing two major and two minor protein isoforms that apparently keep all catalytic residues in common but diverge in the C-terminal part [57,58,59]. The substrate preference of AtMMH-1, as characterized initially, is reminiscent of Fpg and OGG1 with its ability to excise 8-oxoG and discriminate against A opposite to the lesion [57], and both AtOGG1 (see below) and AtMMH-1 (isoform 1) suppress characteristic 8-oxoG-induced G→T transition in a reporter *fpg E. coli* strain [60]. Interestingly, when compared side by side, purified AtMMH-1 is much less active in the removal of 8-oxoG than AtOGG1 [61]; however, 8-oxoG repair activities in *MMH-1* and *OGG1* knockout cell extracts are affected to about an equal degree [26]. Further studies with oligonucleotide and irradiated DNA substrates showed excellent activity by AtMMH-1 against Fapy bases and products of four-electron G oxidation (guanidinohydantoin and spiroiminodihydantoin), but only minor capability for 8-oxoG removal [62]. The structural reason for this is, apparently, the lack of the “capping loop” in the C-terminal domain, which partly forms an 8-oxoG recognition pocket in bacterial Fpg but is much shorter in AtMMH-1 [63]. Notably, the shorter major AtMMH-1 isoform deviates from the longer isoform, starting immediately before this loop and does not rescue the *fpg* phenotype when expressed in *E. coli*, raising questions about the substrate specificity of this protein variant [60].

Besides *A. thaliana*, MMH homologs were partly characterized in sugarcane [64,65] and barrel clover (*Medicago truncatula*) [66]. The sugarcane genome encodes two MMH homologs that are highly conserved (but not identical) in their N-terminal domain and in the C-terminal domain up to the H2TH motif but diverge thereafter; both are ubiquitously expressed and were shown to decrease mutations in the *fpg mutY E. coli* reporter strain [65]. The barrel clover protein was not characterized but, together with OGG1, is upregulated in response to heavy metals and osmotic stress and during seed imbibition, all known to induce oxidative stress [66]. In a related species, alfalfa (*M. sativa*), MMH is upregulated in response to cell exposure to quantum dots, which apparently also cause oxidative stress [67].

After a long history of failures to find eukaryotic orthologs of bacterial Fpg in a pre-genomic era, in 1996, a non-homologous 8-oxoguanine glycosylase, OGG1, was cloned from yeast based on the ability to complement the mutator phenotype of *fpg mutY*-deficient *E. coli* [68] or cross-link specifically to an 8-oxoG:C duplex [69]. The human [70,71,72] and *A. thaliana* orthologs [73,74] followed soon. As the yeast and human proteins, AtOGG1 excises 8-oxoG via a Schiff base intermediate, preferring 8-oxoG:C over 8-oxoG:A pairs, and is capable of rescuing the mutator phenotype of *fpg* or *fpg mutY*-deficient *E. coli* [60,73,74,75]. Besides 8-oxoG, AtOGG1 can remove FapyG, but not other oxidative lesions generated by γ-irradiation of DNA [75]. The *AtOGG1* gene is universally expressed and is induced neither by oxidative stress nor increased 8-oxodGTP caused by a deficiency of AtNUDX1, the only 8-oxodGTPase in *A. thaliana* [73,74,76]. Interestingly, unlike human OGG1, which undergoes extensive alternative splicing and generates 13 protein isoforms [77], only one mRNA and protein are known for AtOGG1.

Transgenic *A. thaliana* plants overexpressing OGG1 show increased seed longevity and resistance to osmotic and heat stress [25]. OGG1 is upregulated in response to the conditions known to induce oxidative stress, such as heavy metals and osmotic stress, and histone deacetylase inhibitor trichostatin A in barrel clover, as well as during seed imbibition in *A. thaliana*, barrel clover, and eggplant [25,66,78,79].

Together, MMH-1 and OGG1 account for the majority of 8-oxoG repair in *A. thaliana,* but some residual activity is still observed in the double knockouts [26]. Its nature remains to be established. The gene that is the last member of the GO system, MutY, is present in plants, but has not been characterized so far.

As for oxidized pyrimidine repair, *A. thaliana* possesses two Nth homologs, NTH1 and NTH2 [80,81], which are rather poorly studied at present. Recombinant NTH1 and NTH2 are bifunctional DNA glycosylases that efficiently release thymine glycol and urea residues from DNA and cleave the backbone by β-elimination. The knockout of both genes has no overt phenotype [81].

## 4. Epigenetic Glycosylases, the Plants’ Unique Toolkit

**Figure 3:**
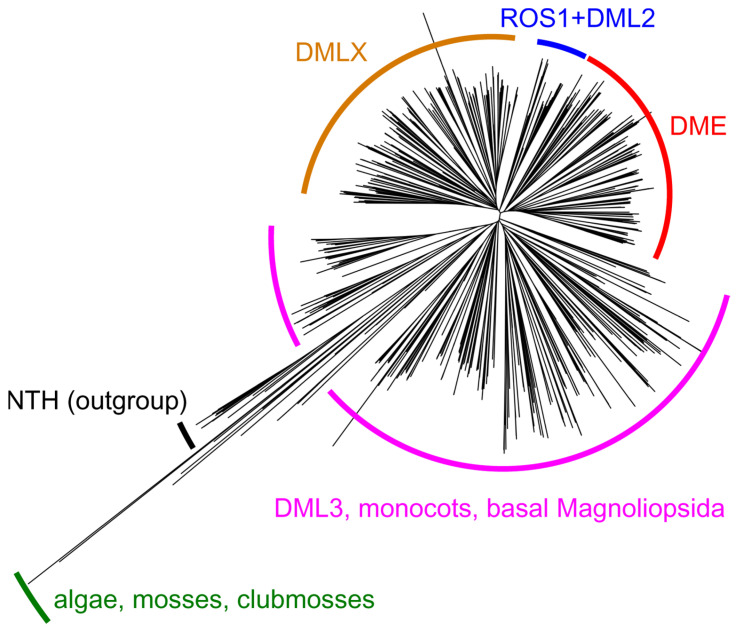
Unrooted tree with 989 plant DML protein sequences. NCBI RefSeq protein database [90] was searched using AtDME, AtROS1, AtDML2, and AtDML3 as queries, limited to the Viridiplantae kingdom. Only the sequences containing the HhH motif, the iron–sulfur cluster, the zinc finger, and the RRM motif were retained. Nineteen plant NTH proteins were taken as an outgroup. The sequences were aligned with MUSCLE [91], and the tree was built with the PHYLIP neighbor-joining method [92], as implemented in UGENE v48 [93]. The tree was visualized using iTOL [94]; the Newick file can be found in the Supplementary Materials (Grin_et_al_Fig_3.nwk).

Gene expression in higher eukaryotes is controlled through several mechanisms, of which epigenetic DNA methylation is especially prominent. In most animals and plants, it relies on a “fifth nucleobase”, 5-methylcytosine (^m^C), serving as a reversible mark that plays an important role in transcription regulation and genome protection from mobile elements [82,83]. Vertebrates overwhelmingly use CpG dinucleotides for this purpose, whereas plants are more flexible, methylating C in CG, CHG, and CHH contexts (H = A, C, or T). In all species that use ^m^C residues as epigenetic marks, they are set on by DNA (cytosine-5)-methyltransferases that either methylate DNA de novo or reinstall ^m^C in hemimethylated DNA. However, the opposite-direction process, active demethylation, employs entirely different mechanisms in animals and plants, albeit both based on BER. In animals, ^m^C is oxidized by TET family dioxygenases first to 5-hydroxymethylcytosine, an epigenetic mark itself, and further to 5-formylcytosine and 5-carboxycytosine; all these derivatives can also be actively deaminated to 5-hydroxymethyluracil, 5-formyluracil, and 5-carboxyuracil, respectively. The oxidized/deaminated bases are excised by TDG and replaced with C through the BER pathway [84,85]. Plants, in contrast, possess DNA glycosylases that directly recognize and excise ^m^C. In 2002, two paralogous genes involved in the epigenetic control in *A. thaliana* were found to encode DNA glycosylases: *DEMETER* (*DME*), a gene responsible for maternal allele derepression of *MEDEA* Polycomb group chromatin remodeler during embryo and endosperm development, and *REPRESSOR OF SILENCING 1* (*ROS1*, also known as *DEMETER-LIKE 1*, *DML1*), an activator of transcription of several endogenous genes acting throughout the life cycle [86,87]. Two more paralogs, termed *DEMETER-LIKE 2* (*DML2*) and *DEMETER-LIKE 3* (*DML3*), were characterized shortly thereafter [86,88,89]. There is a fifth paralogous gene residing on chromosome 3 (At3g47830), which remains uncharacterized, but its predicted protein product lacks several critical functional domains (see below) and may be non-functional. Finally, the analysis of sequenced plant genomes reveals another cluster of homologs (labeled DMLX in Figure 3), distributed in the major groups of dicots. They keep all functional domains of DML proteins but differ in their long, disordered N-terminal tails. No representative of this branch has been studied so far.

A word about the nomenclature is prudent here, since DME-like genes/proteins from different species often bear the name “DML” or “ROS” followed by a number, but are most similar to differently numbered DME-like proteins from *A. thaliana*. DME is the first discovered member of this small family. ROS1 is systematically known as DML1 and can be found under this name in some papers; however, here we keep the name ROS1 to be consistent with the bulk of the literature. DML2 and DML3 in *A. thaliana* have been so named from the moment of their discovery [86]. However, in tobacco, for example, four demethylating glycosylases were described as ROS1, ROS2a, ROS2b, and ROS3, all being closer to AtROS1 than to other DML homologs from *A. thaliana* [95], and of three predicted genes more similar to DME, DML2, and DML3, two are designated “DEMETER-like”, and one, “ROS1-LIKE”. Also, with the expanded number of sequenced plant genomes, it became apparent that true DME homologs are present only in dicots, whereas all homologs from monocots should be grouped with DML3, which appears to be ancestral to the whole family ([96,97] and Figure 3). Therefore, “DME” and “ROS1” proteins in wheat, barley, and rice [98,99,100,101,102] should actually be DML3, but we will keep the original names (in quotation marks) to be consistent with the other publications. We will further refer to the whole group as DML proteins or DML glycosylases.

DME and ROS1 are 5-methylcytosine–DNA glycosylases with a strong preference for ^m^CG dinucleotides [103,104]. Some activity in ^m^CHG and ^m^CHH contexts is also observed, consistent with the known methylation pattern in plants. They can excise T with much lower efficiency, but have no activity on U bases [103]. Both DME and ROS1 possess a Lys–Asp catalytic dyad characteristic of bifunctional HhH superfamily DNA glycosylases and cleave DNA by β- and β,δ-elimination forming a Schiff base intermediate in the process [103,104,105]. Besides DME and ROS1 from *A. thaliana*, recombinant ROS1 from tobacco was partially biochemically characterized [95,106,107].

All DML proteins possess a unique split HhH catalytic domain, and an iron–sulfur cluster, a solitary zinc finger, and a putative RNA recognition motif-containing (RRM) domain [108,109]. They are all grouped in the C-terminal part of the protein, while the N-terminal part shows no homology to other known proteins and is highly variable even within DML proteins. The catalytic domain carries a large insertion of variable length (several hundred residues) between the αB and αC helices of the six-helix barrel domain, but the arrangement of the residues important for catalysis is conserved between DMLs and other HhH glycosylases, such as Nth [109,110]. Interestingly, the N-terminal domain of AtDME is dispensable for correct demethylation of canonical functional gene targets, but its removal impedes demethylation in heterochromatin and causes demethylation of non-specific gene body targets [111].

Of all DML proteins, ROS1 is probably the best investigated in terms of its enzymatic properties. In addition to ^m^C and T, it efficiently removes 5-hydroxyuracil and 5-hydroxymethylcytosine, has moderate activity on 5-fluorouracil, 5-bromocytosine, and 5-bromouracil, and possesses only marginal ability to excise 5-hydroxymethyluracil [105,107,112]. The high activity against 5-hmC is especially intriguing since plants have long been believed to lack this modification in their genomes, but recent advances in mass spectroscopic detection and single-base resolution sequencing revealed its presence in DNA from several plant species [113,114,115]. AtDME and AtDML3 can also excise 5-hmC [116]. With respect to the strand methylation status, ROS1 utilizes both hemimethylated and bimethylated substrates but prefers the former, which may be important to avoid double-strand breaks [105,107]. Damage to the opposite G base adversely affects ^m^C excision, which again may be important to prevent mutagenesis by misincorporation during BER [106]. The enzyme forcibly opens ^m^C:G pairs using a trio of residues (Gln607, Arg903, and Met905) that intercalate into the DNA helix [117]. The iron–sulfur cluster is also necessary for ROS1 and DME activity, and cluster-disrupting mutations, chemical oxidation, or inactivation of the cellular cluster assembly pathway render these enzymes non-functional [118,119,120,121,122]. The RRM motif is essential for DNA binding and ^m^C removal in vitro and male fertility in vivo, despite being missing in all other HhH glycosylases [97,123]. Like many HhH glycosylases, ROS1 is a slow-turnover enzyme that binds its AP site product with high affinity [105]. The search for mC by ROS1 in long DNA targets proceeds through non-specific DNA binding and one-dimensional diffusion along DNA, a behavior characteristic of many DNA glycosylases, and depends on the presence of the long N-terminal domain [117,124,125]. ROS1 is sumoylated by SIZ1 SUMO E3 ligase, which stabilizes ROS1 and enhances demethylation of many of its targets [126]. In vivo, ROS1 appears to function as part of a multiprotein complex, also involving a WD40 domain-containing protein RWD40, a methyl-DNA binding protein RMB1, and a zinc finger and homeobox domain protein RHD1 [127]. ROS1 is anchored to the complex through the interaction of its N-terminal tail with RWD40, whereas RMB1 acts as a ^m^C reader targeting the complex to methylated genome regions [127].

Despite the presence of RRM domains, it is not clear whether DML proteins are guided to their site of action by any RNA. In vivo, the proper targeting of ROS1 depends on ROS3, a small RNA-binding protein, but physical interaction between ROS1 and ROS3 has not been demonstrated [128]. Demethylation by DMLs requires the glycosylase interaction with the H1 linker histone and prior chromatin opening by the MBD7 methyl-CpG-binding protein and IDM1/IDM2 histone H3 acetyltransferases [129,130,131,132]. ROS1 directly binds H3 histone through the RRM and this interaction is regulated by specific histone tail modifications in vitro [123], whereas in chromatin, ROS1 targets are enriched in H3K18ac and H3K27me3, and depleted of H3K9me2 and H3K27me1 [133].

The biochemistry of DML2 and DML3 has received much less attention, but both enzymes seem to be similar to DME and ROS1 in terms of the substrate specificity and reaction mechanism, although DML2 lacks thymine-excising activity [88,89]. It remains to be seen whether they have any specialized roles.

DME, so far, seems to act only in the regulation of plant development. Unlike mammals, in which a global wave of demethylation occurs immediately after fertilization and then new ^m^C marks are laid out, plants experience more local methylation reconfiguration in the early zygote [134,135]. DME supports temporally and spatially restricted expression of maternal origin alleles normally silenced in the rest of the plant and in other developmental phases, such as *MEA*, *FLOWERING WAGENINGEN* (*FWA*), *FERTILIZATION INDEPENDENT SEED 2* (*FIS2*), or *FLOWERING LOCUS C* (*FLC*) in *A. thaliana* [88,136,137,138,139]. DME also accounts for development-associated demethylation and activation of many mobile elements [140]. Both DME and ROS1 are required for proper pollen tube growth and orientation regulating a network of pollen tube signaling through active demethylation and gene activation [141]. In vegetative tissues, the only known role of DME is in regulation of pathogen defense genes [142], which is in common with ROS1, DML2, and DML3 [143].

Other DML proteins are also partially involved in development regulation. For example, in wheat (eleven homologs), barley (three homologs), and rice (six homologs), “DME” and “ROS1a” derepress genes coding for prolamins, the main storage proteins, during seed development [99,102]. In rice, both paternal and maternal loss-of-function alleles of “ROS1a” are lethal [98], and at least three DML glycosylases participate in demethylation in the gametes and the zygote [101]. In sorghum, two DML homologs are markedly upregulated in callus cultures, indicating the involvement of active demethylation in cell (de)differentiation processes [144]. The efficiency of organ regeneration from explants was shown to correlate with DME, ROS1, and DML2 levels in rock violet (*Boea hygrometrica*) [145].

ROS1, DML2, and DML3 primarily regulate gene expression in somatic tissues. Mutant *ros1 A. thaliana* plants show a dramatic increase in methylation of some endogenous promoters, such as *RD29A*, *FWA,* or *EPF2,* while overexpression of ROS1 induces their demethylation and activation [104,146,147]. Importantly, ROS1 activity is required for methylation and suppression of transposons of several types (*GP1*, *LINE1-4*, *SN1*, *MU1*) in plant cells [146], and ROS1/DML2/DML3-targeted regions are enriched in repeats that are considered the remnants of transposons [148]. One of the DML proteins in rice has been shown to demethylate and activate the *Tos17* retrotransposon, a *Copia* family element widely used for experimental insertion mutagenesis in rice [149]. Rice DMLs also participate in differential methylation during generative cells production and early plant development [150] and are upregulated by heavy metals stress [151]. The tomato genome encodes four DML proteins, one of which, most homologous to ROS1, is upregulated in ripening fruit, and its suppression delays ripening due to the hypermethylation of promoters of several genes involved in the process [152,153]. The upregulation of DML proteins correlating with methylation changes has been demonstrated in rice for spaceflight [154]. *A. thaliana* requires ROS1, DML2, and DML3 to mount an immune response to *Pseudomonas syringae* infection [143], while in potato, ROS1 is elevated in response to β-aminobutyric acid, a pathogen signaling molecule that primes plant cells to fungal infection [155].

Despite an apparently wider range of processes and targets in comparison with DME, the methylation control by ROS1 seems to be limited to a few loci. Although ROS1/DML2/DML3-targeted regions contain many methylation-directing siRNA targets, at least ROS1 seems to remove methyl labels whose origin is not limited to RNAi-dependent pathways [148,156]. Strikingly, the global methylation level is not changed even in triple *ros1 dml2 dml3* mutants [88]. Bisulfite sequencing reveals that ROS1, DML2, and DML3 display a degree of specificity for the demethylated loci, but in most cases it is not absolute [88,139]. Quadruple *dme ros1 dml2 dml3* mutants lacking all DML proteins in somatic tissues can be obtained by central cell-specific ectopic expression of *DME* and display global hypermethylation and loss of tissue-specific differential methylation [139].

Interestingly, expression of *ROS1* itself is *positively* controlled by methylation: a helitron transposon is present in front of the ROS1 promoter and suppresses transcription, whereas a specific “methylation monitoring sequence” in between can be methylated to uncouple the helitron from the promoter and boost ROS1 expression [157,158]. *DML* genes are also regulated by miRNA in development and during abiotic stress, as documented in *A. thaliana*, barley, and red sage (*Salvia miltiorrhiza*) [100,159,160].

Although the full spectrum of plant cell responses involving DML glycosylases has not yet been elucidated, it is clear that these proteins orchestrate gene activity in development and integrate many responses to various kinds of abiotic stress. In dicots, DME seems to be strictly dedicated to developmental regulation, while other DMLs participate both in development and in the stress response. In monocots, such specialization is not yet clear. A concept on “stress memory” has been proposed, in which the methylation status changes in response to abiotic stress can be imprinted and stably maintained for some time in plant development [161], or even in a trans-generational manner [162], through the balanced action of DML proteins and the methylation apparatus.

On a more practical side, the expression of transgenes in plants is often regulated by methylation-sensitive elements, such as cauliflower mosaic virus 35S promoter, and regulation of ROS1 was proposed as a way to drive or silence the expression of transgenes [163]. DML glycosylases are also promising tools for epigenetic status manipulation in non-plant cells: transfecting DME or ROS1 into human cells causes global loss or the ^m^C epigenetic label [107,164,165], and fusions of ROS1 with specific DNA-addressing domains induce targeted DNA demethylation and gene activation [166,167]. However, DME overexpression in human cells induces DNA damage, the stress response, interferon production, S phase arrest, and ultimately cell death [164]. Interestingly, non-targeted demethylation by ROS1 in cancer cells also causes methylation gain, partially reversing the methylome pattern to that characteristic of normal cells [165].

## 5. Unexplained Diversity of Alkylation Damage Glycosylases

The major products of electrophilic addition removed by BER are purines alkylated at *N*3 and *N*7 positions, whereas products of alkylation at exocyclic groups and other ring nitrogens (N1 of purines, N3 of pyrimidines) are repaired by Ada-like alkyltransferases and AlkB-like dioxygenases without base excision [168,169,170]. DNA glycosylases from several structural superfamilies are capable of alkylation damage removal. In *E. coli*, the AlkA protein, which belongs to a wide, functionally diverse HhH superfamily, is induced by alkylation stress, and removes *N*3- and *N*7-alkylpurines and 1,*N*^6^-ethenoadenine (εA). Another *E. coli* alkylpurine–DNA glycosylase, Tag, is constitutive, and its substrate specificity is limited to *N*3-alkyladenine; structurally, it is sometimes also grouped with HhH proteins, but outside of the namesake helix–hairpin–helix motif, it is very different from the rest of the superfamily. In human cells, MPG protein (also known as AAG or APNG) removes 3-methylpurines, hypoxanthine, and εA and belongs to a completely different structural family, resembling the C-terminal domain of methionyl-tRNA^fMet^ formyltransferase [171,172]. Moreover, some bacteria (but not *E. coli*) possess DNA glycosylases belonging to the HEAT repeat domain and winged-helix domain superfamilies and specific for alkylated purines or purine adducts with some natural genotoxic antibiotics [173,174].

In vertebrates, the primary endogenous source of DNA alkylation is stray reactions with metabolic methyl group donors, mainly *S*-adenosyl methionine [3]. Many plants, however, synthesize DNA-alkylating secondary metabolites that are either electrophiles or are metabolized to electrophilic species and, thus, may put the plant’s own genome under threat [12,14]. Thus, the problem of alkylation damage repair can be more acute in plants. Admittedly, many bulky adducts with electrophilic species may be removed by nucleotide excision repair [175] or mismatch repair [176], and ring-alkylated purine nucleotides can decay spontaneously [177], yet BER still plays an important role in fixing DNA damage from these lesions.

**Figure 4:**
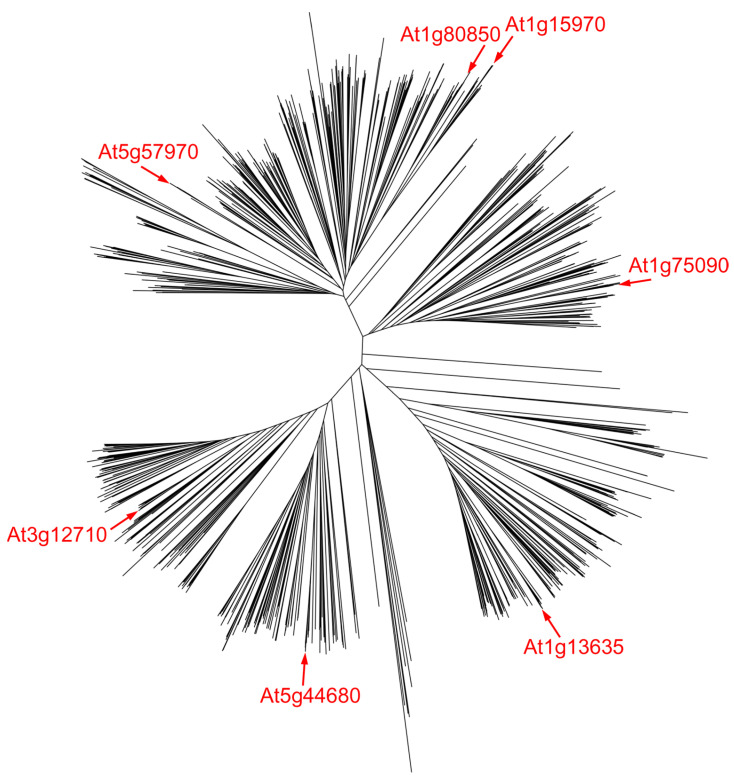
Unrooted tree with 1064 plant TAG protein sequences. NCBI RefSeq protein database [90] was searched using *E. coli* Tag and seven *A. thaliana* TAG homologs (marked red, Table 1) as queries, limited to the Viridiplantae kingdom. The sequences were aligned with MUSCLE [91], and the tree was built with the PHYLIP neighbor-joining method [92], as implemented in UGENE v48 [93]. The tree was visualized using iTOL [94]; the Newick file can be found in the Supplementary Materials (Grin_et_al_Fig_4.nwk).

Plant cells excise both 3-mA and 7-mG from methylated DNA [178]. Most unfortunately, the mechanisms of this repair received very little attention, and only today, when many plant genomes have been sequenced, do we realize that they are exquisitely enriched in homologs of alkylpurine glycosylases. Gene duplication in plants followed by specialization of paralogs are not unusual [179], and some BER genes have apparently undergone such events, as the DML proteins discussed above can attest. Among other DNA glycosylases, a UNG pseudogene (At2g10550) arising from duplication and then disrupted by two transposon insertion events is found in the *A. thaliana* genome [42]. Compared with other branches of life, TAG genes are highly overrepresented in plant genomes [180]; *A. thaliana* possesses seven TAG homologs, some of which appear to result from ancestral duplications, while others appeared recently (Figure 4). On top of that, the *A. thaliana* genome codes for three AlkA homologs and one MPG homolog. The last one is actually the only plant alkylpurine–DNA glycosylase characterized in any detail: a cDNA from an *A. thaliana* library was cloned based on its functional ability to complement the methyl methanesulfonate sensitivity in an *alkA tag E. coli* strain [181]. Although the protein was not purified, extracts of *E. coli* cells harboring the plasmid with the gene insert excised 3-mA, but not 7-mG [181]. The expression of maize MPG is induced by UV and zeocin (a radiomimetic drug) treatments [182], but the significance of this finding is uncertain since neither agent is alkylating.

Both targeted and high-throughput experiments show that all alkylation damage glycosylases are expressed at some stages in the plant life cycle, so it is unlikely that the homologs detected at the genetic level represent pseudogenes [183,184]. It remains to be seen what their biologically relevant substrates are and whether some of them play organ- and development-specialized roles.

## 6. AP Site Cleavage and 3′-End Processing

All three types of DNA glycosylase products, namely AP site, 3′-terminal unsaturated aldehyde, and 3′-terminal phosphate, need further processing to expose a 3′-OH end that can be extended by DNA polymerases (Figure 1 and Figure 2). In *E. coli*, all these reactions are catalyzed by either of two AP endonucleases, exonuclease III (Xth, belonging to the Exonuclease–Endonuclease–Phosphatase (EEP) structural superfamily) and endonuclease IV (Nfo, triose phosphate isomerase barrel structural superfamily) [185]. Xth is a constitutive protein, while Nfo is induced by oxidative stress but is still present at the level of ~10% of Xth in uninduced cells. Higher eukaryotes possess two Xth homologs, APE1 and APE2. The former is the major AP endonuclease in human cells and can also remove 3′-terminal sugar remnants, but lacks the 3′-phosphatase activity, and these ends are cleaned by PNKP [186,187]. APE2 is predominantly a 3′→5′ exonuclease, rather than an AP endonuclease, and is involved in end resection at single-strand breaks in several DNA repair pathways, including BER [188].

Three *A. thaliana* enzymes belonging to the EEP superfamily, AtAPE1L, AtARP, and AtAPE2, show an AP endonuclease activity [189,190]. Additionally, there is a functional equivalent of PNKP, AtZDP (see below). APE2 is a closer homolog to human APE2 and, similar to its human counterpart, possesses only a modest AP endonuclease activity, but strong 3′→5′-exonuclease activity [190,191]. Of the remaining two, APE1L is apparently more important, since *ape1L ape2* and *ape1L zdp* double knockout seeds are inviable, whereas those of the *arp ape1L* and *arp ape2* genotype survive [190,192,193]. APE1L also eagerly removes 3′-terminal unsaturated aldehyde moieties generated by DNA glycosylases that carry out β-elimination and the 3′-terminal phosphate, the product of β,δ-elimination [190].

APE1L interacts with ROS1 forming a ternary complex on DNA, but does not displace ROS1 from the reaction product [190]. This distinguishes APE1L from hAPE1, which stimulates many DNA glycosylases by disrupting the tight enzyme–product complexes [194]. Methylation patterns are severely disrupted in *ape1L* and *ape1L zdp* knockout cells [190].

ARP proteins from *A. thaliana*, wheat, and sugarcane demonstrate the AP endonuclease activity [190,195,196,197] and, in addition, can cleave DNA 5′ of some damaged nucleotides, such as α-deoxyadenosine [196]. The 3′→5′-exonuclease, 3′-phosphodiesterase, and 3′-phosphatase activities are somewhat controversial; AtARP was reported to lack [190] and to possess them [196]. ARP is involved in the post-incision steps during U and 8-oxoG repair [195,198]. *A. thaliana arp* plants show sensitivity to methyl methanesulfonate and *tert*-butyl hydroperoxide, but not to H_2_O_2_ [196].

In addition to their AP endonuclease activity, mammalian APE1 proteins possess so-called “redox activity”, reactivating many oxidized transcription factors [199]. ARP has been reported to possess redox activity [189]; however, its mechanism is unclear since no equivalent of the redox-active Cys65 residue of hAPE1 is found in AtARP. Recently, the redox nature of transcription factor reactivation has been questioned [200,201].

The APE-less BER branch in plants relies on the zinc finger DNA 3′-phosphatase (ZDP), rather than on PNKP as in mammals [198]. AtZDP efficiently removes the 3′-phosphate from a gapped substrate, but lacks the kinase domain present in human PNKP and, thus, has no 5′-kinase activity [198,202]. In addition, instead of the forkhead-associated domain of PNKP responsible for XRCC1 binding [203], ZDP is equipped with three PARP-like zinc fingers, which may serve as a nick sensor [202,204,205]. In addition to MMH-1, an efficient catalyst of β,δ-elimination, ZDP assists the processing of intermediates formed by OGG1 and DMLs, which also produce a fraction of product with a 3′-terminal phosphate [26,103,105,198,206]. Genetic data show that APE2 is epistatic with ZDP in DML-associated demethylation, but the phosphatase activity of APE2 is weak, and the repair probably depends on its 3′→5′-exonuclease activity [191]. In *zdp ape2* double knockouts, 3′-blocked BER intermediates accumulate and cause proliferation and development defects partially rescued by abrogation of MMH glycosylase/β,δ-lyase or of SOG1, a plant-specific transcriptional regulator acting in a break-sensing ATR pathway [28]. Thus, APE1L, APE2, and ZDP all appear to participate in 3′-end cleaning in plants.

DNA glycosylases/AP lyases can also process AP sites, formed either by spontaneous depurination or by other DNA glycosylases. Until recently, this reaction was considered rather irrelevant biologically because the processing of the 3′-ends would still require AP endonucleases or 3′-phosphatases. However, recently the repair of damage induced by methyl methanesulfonate (MMS) in *A. thaliana* was shown to depend on the sequential action of MMH-1 and ZDP [177]. MMS predominantly produces *N*7-methylguanine, which either depurinates spontaneously or is excised by the strictly monofunctional alkylpurine glycosylases, producing an AP site. It has been suggested that, at least in plants, ARP and/or APE1L may be predominantly involved in the repair of AP sites formed by DNA glycosylases, whereas AP lyases contribute significantly to the repair of spontaneously formed AP sites [177,207].

## 7. Downstream Events in Plant BER

### 7.1. DNA Polymerases

*A. thaliana* cell extracts are proficient in both short- and long-patch BER [41,195,208]. In a major departure from the human paradigm, plants lack homologs of POLβ, which initiates repair DNA synthesis in both short- and long-patch BER. Nevertheless, POLλ is present and functional, possessing both polymerase and dRP lyase activities [202,209,210]. Its expression is induced by MMS, UV and white light, and salt stress [210,211,212]. As in human cells, AtPOLλ is involved in non-homologous end joining, and likely participates in nucleotide excision repair of UV-B damage [211,213]. Moreover, similar to the human system [214], AtPOLλ shows the ability to incorporate a correct C opposite to 8-oxoG, making it a good candidate for the GO system polymerase [215]. All this said, direct evidence of POLλ participation in plant BER, such as reconstitution experiments or the establishment of the full BER cycle in knockout plants, is lacking at the moment.

There definitely is a possibility that other plant DNA polymerases, whose main role is in replication, translesion synthesis, or other DNA repair pathways, also participate in BER. In addition to POLλ, plant genomes code for homologs of human DNA polymerases γ and θ (Family A), α, δ, ε, ζ (Family B), η, κ, and REV1 (Family Y). In the literature, for historical reasons, two *A. thaliana* POLγ homologs are often referred to as homologs of *E. coli* DNA polymerase I (PolI), albeit they are actually closer to POLγ. Mutations in the small subunit of POLδ, POLD2, are hypersensitive to MMS, which may suggest POLδ involvement in BER [216]. The POLγ/PolI homologs have been shown to participate in BER in mitochondria and chloroplasts (see below).

### 7.2. Flap Endonuclease

Flap endonuclease, FEN1, is a key enzyme in long-patch BER in vertebrates, removing the strand displaced by a DNA polymerase once it progresses beyond the replacement of a single nucleotide [217,218]. FEN1 is not a specific BER enzyme, its main role being the removal of Okazaki fragments during lagging-strand replication [218]. *A. thaliana* FEN1 was independently cloned by two groups in 2016, as a suppressor of transgene silencing and a regulator of light-dependent root and hypocotyl elongation [219,220]. Biochemically, AtFEN1 is similar to hFEN1, but lacks a 5′→3′-exonuclease activity, and the mutant plants with a splicing defect show sensitivity to MMS and UV, but are still viable, unlike complete knockouts [219,220]. FEN1 homologs from cauliflower and rice (two paralogs) were purified or cloned and characterized, showing essentially the same properties, except the presence of the 5′→3′-exonuclease activity in one of the rice proteins [221,222,223,224]. The second rice homolog might be non-functional, since it does not complement the *rad27* yeast strain deficient in FEN1 [224].

### 7.3. DNA Ligases

In vertebrates, two DNA ligases participate in different BER branches: Lig IIIα completes short-patch BER, and Lig I works in the long-patch subpathway [217]. As with FEN1, Lig I’s main role is in replication. Lig IIIα is absent from plants, and Lig I (AtLIG1 in *A. thaliana*) seems to be the only DNA ligase acting in plant BER [195,225]. Genetic evidence suggests that LIG1 works in the same pathway as MBD4, DME, and ROS1 [49,226,227]. *A. thaliana* also possesses a second homolog of Lig I (At1g49250), which remains uncharacterized at the moment.

### 7.4. XRCC1

XRCC1, the adaptor protein, orchestrates the coordinated transfer of repair intermediates between sequential BER enzymes in higher eukaryotes [228,229]. Plant XRCC1 homologs have been characterized from *A. thaliana* [230] and rice [231]. Notably, AtXRCC1 is about twice as short as the human protein and lacks both the N-terminal domain and the second BRCT domain that forms the dimerization interface [232,233]. Nevertheless, plant XRCC1 proteins seem to be functionally equivalent to hXRCC1, at least with respect to BER: they bind DNA forming a scaffold that brings together the upstream actors (at least ROS1 and ZDP) and stimulate their activity both as purified proteins and in cell extracts [230]. Co-localization of APE1L, ZDP, and ROS1 observed in living cells [190] may be due to their assembly on the XRCC1 scaffold. *A. thaliana* plants deficient in XRCC1 are viable but radiosensitive [234].

### 7.5. Poly(ADP-Ribose) Polymerases

Poly(ADP-ribose) polymerases are multifunctional proteins sensing single- and double-strand breaks and initiating post-translational modification of many proteins, including themselves by mono- and poly(ADP-ribose) [235,236]. In humans, the PARP family comprises 17 members, the best studied of which is PARP1, and the role in DNA repair has also been shown for PARP2 and PARP3. The main function of PARPs in BER seems to be the reorganization of chromatin at single-strand breaks formed after AP endonuclease action (or spontaneously), clearing the site of action for further BER steps. In contrast, *A. thaliana* possesses only three PARPs; in the literature, two of them are somewhat non-discriminately named either PARP1 or PARP2. AtPARP1 (At2g31320) seems to be the primary poly(ADP-ribose) polymerase in DNA damage response, while AtPARP2 (At4g02390) is more active as a recombinant protein in vitro, but apparently plays a back-up role when assayed in cell-free extracts [237,238]. AtPARP1 and AtPARP2 knockouts are hypersensitive to MMS, and the expression of both genes is induced by zeocin [239]. AtPARP3 (At5g22470) is highly expressed in seeds and is required for seed storability, yet it is not induced by genotoxic stress and purified recombinant AtPARP3 shows no activity [238,239,240]. Overall, PARP1 and PARP2 seem to participate in plant BER.

## 8. BER in Plant Mitochondria and Chloroplasts

BER in organelles is organized along with the general BER scheme, but has some important details. The best-studied example of organellar BER is provided by mammalian mitochondria [241,242]. The majority of DNA glycosylases, as well as APE1, have been experimentally shown to localize in mitochondria, or their mitochondrial localization signals can target reporter proteins to mitochondria, or at least have predicted mitochondrial isoforms. POLγ is the main BER polymerase in mitochondria, and the ligation step depends on the mitochondrial isoform of Lig IIIα.

*A. thaliana* and potato tuber mitochondria can support the full BER cycle for U [243,244], and complete BER of 5-hydroxyuracil was detected in mitochondrial extracts from potato [244]. Unlike human *UNG* gene, which produces mitochondrial UNG1 and nuclear UNG2 isoforms with different N-terminal tails via alternative transcription initiation and splicing, only one AtUNG mRNA and protein exist; however, the first 174 residues of AtUNG have been shown to target an EGFP reporter to mitochondria [243]. Interestingly, UNG and, to a lesser degree, AP endonuclease activity is partly localized in the mitochondrial membrane fraction [243]. This is reminiscent of the membrane localization of the mitochondrial fraction of human UNG and OGG1 [245,246]. An interesting but still untested possibility is that the inner membrane, which both hosts the electron transport chain and is tightly associated with mitochondrial DNA [247], is the principal site of both damage and its immediate repair in this organelle. However, as with the nuclear genome, *ung A. thaliana* plants do not accumulate mutations in mitochondrial DNA, and the same holds for *mmh* and *ogg1* plants, suggesting the presence of alternative repair mechanisms, most likely mismatch repair [44,248]. UNG activity was also partially purified from maize mitochondria [38], and mitochondria from Brazilian pine (*Araucaria angustifolia*) contain activities of UNG, MMH, and AP endonuclease [249]. Pol I/POLγ homologs, characterized from *A. thaliana*, rice, and tobacco [250,251,252,253,254], carry out the repair synthesis step. Notably, one of these homologs, PolIB, carries an insertion in the polymerase domain providing it with the dRP lyase activity, which is absent from other Family A DNA polymerases [254]. Based on the observed error rate, which is ~10-fold higher for AtPolIB than for AtPolIA, and drawing parallels with the low fidelity of POLβ [255], one can surmise that PolIB is a repair polymerase, whereas PolIA is better suited for a role in organellar replication [256]. The mitochondrial isoform of Lig I completes the repair.

In comparison, BER in chloroplasts has received less attention. Regarding the initial BER stages, UNG activity was detected in maize chloroplasts [257] and AtNTH1, AtNTH2, and AtARP were found to be localized in chloroplasts [81]. Both short- and long-patch BER has been observed in chloroplasts, and the same Pol I/POLγ-like DNA polymerases participate in it [251,253,254]. The nature of the ligase remains a mystery, since, at least in *A. thaliana*, Lig I is targeted to nuclei and mitochondria, but not to chloroplasts [258,259]. The overall organization of BER in chloroplasts is most likely similar to nuclear and mitochondrial BER, but many crucial details are still missing.

## 9. Conclusions

Although the principal mechanism of base excision DNA repair in plants is the same as in vertebrates, bacteria, and yeast, the evolutionary history and specific lifestyle endowed plants with their unique repair features. The most obvious and seemingly the most important of those is the existence of epigenetic DNA glycosylases, subverting the genome protection machinery for the ends of gene activity regulation. No equivalent of this system is known outside of the Viridiplantae kingdom; the animal TETs/TDG active demethylation pathway apparently evolved independently. Another striking feature of plant BER, still virtually unstudied, is the high diversity of DNA glycosylases homologous to proteins repairing alkylation damage in bacteria. Elucidating their specificity and biological roles might shed light on the unique kinds of genotoxic stress to which plants are exposed as compared to other living things. Processing of DNA glycosylase reaction products, namely AP sites and dirty 3′-ends, also seems to vary between plants on the one hand and humans and bacteria on the other hand, and relies more on AP lyases and 3′-phosphatases in plants, although there is still no clear picture of distinct subpathways following the action of different glycosylases. Finally, chloroplasts, the most distinctive plant organelle, are an exceptionally important source of DNA damage, yet the repair of the chloroplast genome has been barely addressed so far. Understanding the biochemistry of plant DNA repair, especially in the key crop species, such as rice, wheat, vegetable cultures, etc., should advance our ability to breed or engineer new varieties with increased robustness to abiotic stress.

## Figures and Tables

**Table 1:** Principal BER proteins in *E. coli*, humans, and *Arabidopsis*.

Enzyme	*E. coli*	Human	*A. thaliana*
Uracil–DNA glycosylases	Ung	UNG	At3g18630 (AtUNG)
	Mug	TDG	
		SMUG1	
Endonuclease III	Nth	NTHL1	At2g31450 (AtNTH1)
			At1g05900 (AtNTH2)
A:oxoG-specific adenine–DNA glycosylase	MutY	MUTYH	At4g12740 (AtMYH)
8-oxoguanine–DNA glycosylase	–	OGG1	At1g21710 (AtOGG1)
Bacterial alkylpurine–DNA glycosylase I	Tag	–	At1g13635
			At1g15970
			At1g75090
			At1g80850
			At3g12710
			At5g44680
			At5g57970
Bacterial alkylpurine–DNA glycosylase II	AlkA	–	At1g19480
			At1g75230
			At3g50880
Human alkylpurine–DNA glycosylase	–	MPG	At3g12040 (AtMAG)
Methyl-CpG-specific uracil–DNA glycosylase	–	MBD4	At3g07930 (AtMBD4)
Formamidopyrimidine–DNA glycosylase/endonuclease VIII	Fpg		At3g07930 (AtMMH)
	Nei	NEIL1	
		NEIL2	
		NEIL3	
Epigenetic 5-methylcytosine–DNA glycosylases	–	–	At5g04560 (AtDME)
			At2g36490 (AtROS1)
			At3g10010 (AtDML2)
			At4g34060 (AtDML3)
			At3g47830
AP endonucleases, EEP superfamily	Xth	APE1	At2g41460 (AtARP)
			At3g48425 (AtAPE1L)
		APE2	At4g36050 (AtAPE2)
AP endonucleases, TIM barrel superfamily	Nfo	–	–
3′-phosphatase	–	PNKP	At3g14890 (AtZDP)
DNA polymerases, Family A	PolI	POLγ	At1g50840 (AtPolIA)
			At3g20540 (AtPolIB)
DNA polymerases, Family X	–	POLβ	
		POLλ	At1g10520 (AtPOLλ)
Flap endonuclease	PolI	FEN1	At5g26680 (AtFEN1)
DNA ligase	LigA	Lig I	At1g08130 (AtLIG1)
			At1g49250
		Lig IIIα	
BRCT domain scaffold protein	–	XRCC1	At1g80420 (AtXRCC1)
Poly(ADP-ribose) polymerases	–	PARP1	At2g31320 (AtPARP1)
		PARP2	At4g02390 (AtPARP2)
		PARP3	At5g22470 (AtPARP3)

## Data Availability

All data are contained in the paper and Supplementary Materials.

## Supplementary Materials

The following supporting information can be downloaded at: https://www.mdpi.com/article/10.3390/ijms241914746/s1.
